# The Will Adapts, the Mind Stays Sharp, but the Body Keeps Score: Lifespan Unpaid Caregiving Trajectories and Health

**DOI:** 10.1093/geroni/igaf122.1646

**Published:** 2025-12-31

**Authors:** Janecca Chin, I-Fen Lin

**Affiliations:** University of Missouri, Columbia, Missouri, United States; University of Missouri, Columbia, Missouri, United States

## Abstract

Unpaid caregiving can have lasting health consequences. While extensive research has examined the link between caregiving and health, much of it treats caregiving as an isolated event rather than a cumulative life course process. This study adopts a life course perspective to examine how lifespan caregiving trajectories—defined by age at caregiving onset, duration, recurrence, and overlapping responsibilities—shape later-life health across physical, psychological, and cognitive domains. Using data from the Health and Retirement Study and its Life History Mail Survey, latent class analysis identified four distinct caregiving trajectories, and their health outcomes were compared with those of non-caregivers. Multivariable logistic and multinomial regression models assessed the link between caregiving and later-life health. Among women, Early Enduring and Immersed Caregivers (early and prolonged caregivers) exhibited worse physical health than non-caregivers (AME = +0.27 and +0.43 chronic conditions, p < .001). However, depressive symptoms were comparable, except among Immersed Caregivers who reported more distress than non-caregivers (AME = +0.24, p < .05). In contrast, caregiving appeared protective against cognitive impairment, as Common and Recurrent Caregivers (short-term or intermittent caregivers) had lower impairment risks than non-caregivers (AME = -0.06 and -0.05, p < .01). Among men, caregiving had fewer health consequences. Findings challenge one-dimensional portrayals of caregiving, instead highlighting its trajectory-based and multidimensional health effects. While moderate lifespan caregiving may not compromise well-being, the most intensive caregivers—particularly women—face enduring physical health risks. These findings underscore the need for interventions that support high-risk caregivers while recognizing the cognitive benefits of caregiving.

